# Perinatal Overnutrition Exacerbates Adipose Tissue Inflammation Caused by High-Fat Feeding in C57BL/6J Mice

**DOI:** 10.1371/journal.pone.0121954

**Published:** 2015-04-02

**Authors:** Brandon D. Kayser, Michael I. Goran, Sebastien G. Bouret

**Affiliations:** 1 Human and Evolutionary Biology Program, Department of Biological Sciences, Dornsife College of Letters, Arts and Sciences, University of Southern California, Los Angeles, California, United States of America; 2 Department of Preventive Medicine, Keck School of Medicine, Childhood Obesity Research Center, University of Southern California, Los Angeles, California, United States of America; 3 Developmental Neuroscience Program, The Saban Research Institute, Children’s Hospital Los Angeles, University of Southern California, Los Angeles, California, United States of America; University of Kansas Medical Center, UNITED STATES

## Abstract

Obesity causes white adipose tissue (WAT) inflammation and insulin resistance in some, but not all individuals. Here, we used a mouse model of early postnatal overfeeding to determine the role of neonatal nutrition in lifelong WAT inflammation and metabolic dysfunction. C57BL/6J mice were reared in small litters of 3 (SL) or normal litters of 7 pups (NL) and fed either regular chow or a 60% high fat diet (HFD) from 5 to 17 weeks. At weaning, SL mice did not develop WAT inflammation despite increased fat mass, although there was an up-regulation of WAT *Arg1* and *Tlr4* expression. On HFD, adult SL mice had greater inguinal fat mass compared to NL mice, however both groups showed similar increases in visceral fat depots and adipocyte hypertrophy. Despite the similar levels of visceral adiposity, SL-HFD mice displayed greater impairments in glucose homeostasis and more pronounced hepatic steatosis compared to NL-HFD mice. In addition, WAT from SL mice fed a HFD displayed greater crown-like structure formation, increased M1 macrophages, and higher cytokine gene expression. Together, these data suggest that early postnatal overnutrition may be a critical determinant of fatty liver and insulin resistance in obese adults by programming the inflammatory capacity of adipose tissue.

## Introduction

The prevalence of overweight and obesity has increased at an alarming rate in countries that have adopted a Western lifestyle, which includes overconsumption of energy-rich and nutrient-poor food [1,2]. Such lifestyle changes also affect children, which raises major health concerns because obese children and adolescents are more likely to become obese adults [3]. Obesity is the primary risk factor for the development of type 2 diabetes by causing inulin resistance, which results in a greater demand of the pancreas to secrete insulin and eventually β-cell failure in susceptible individuals [4]. However, obesity is not always sufficient to cause insulin resistance, as 30–40% of individuals with a Body Mass Index greater than 35kg/m^2^ have normal insulin sensitivity determined by the hyperinsulinemic-euglycemic clamp [5]. Differences in fat distribution [6], ectopic fat deposition [7], and inflammation [8,9] may determine whether an obese individual becomes insulin resistant. Identifying the environmental determinants and biological processes of these physiologic mediators is critical to understanding the pathophysiology of obesity-induced insulin resistance.

During the past few decades, evidence has accumulated that suggests that alterations in the perinatal environment can substantially contribute to deleterious metabolic outcomes in the developing offspring. In particular, epidemiological and animal studies have revealed that changes in the hormonal and nutritional environments during critical periods of development may increase the susceptibility for the development of obesity later in life. A primary importance has been given to the nutritional environment before birth in part because of epidemiological and animal studies that demonstrated that severe undernutrition during pregnancy results in adult metabolic disturbances in the offspring [10,11]. However, the early *postnatal* environment also contributes to obesity and metabolic disease risk in adulthood [12,13]. For example, offspring from dams fed a high fat diet (HFD) during the suckling period, but not during gestation, develop leptin resistance, glucose intolerance, and impaired β-cell innervation that persist into adulthood [14,15]. Another valuable model to study postnatal overfeeding is the small litter size model. Pups raised in small litters (SL) have increased energy intake and subsequently gain more weight before weaning [13]. SL rodents remain overweight and hyperinsulinemic into adulthood [16–18], and also develop more pronounced insulin resistance and hepatic steatosis when fed a high-fat diet (HFD) [16,19,20]. This latter observation suggests that early-life nutrition may determine whether an obese individual subsequently develops insulin resistance or remains metabolically normal. However, the biological processes linking perinatal overnutrition and insulin resistance in adulthood remain largely unknown, but might involve adipose tissue inflammation

Obesity in adults is associated with chronic low-grade inflammation that predisposes to insulin resistance [21]. White adipose tissue (WAT), in particular, contributes to this state of metabolic inflammation or “metainflammation,” and undergoes considerable changes in leukocyte composition and cytokine and adipokine production in obesity [21]. Adipose tissue macrophages are central contributors to metainflammation [22], whereby obesity leads to an influx of proinflammatory type 1 macrophage (M1) that overcome the decreasing proportion of resident and anti-inflammatory type 2 macrophages (M2) [23–25]. The recruited M1 macrophages secrete an array of cytokines and chemokines that perpetuate inflammation and impair adipocyte function [26–28]. Increased WAT macrophage content has been suggested to explain, at least in part, the discrepancy between metabolically normal and abnormal obesity in humans [8,9], indicating that environmental factors that modify metainflammation are important drivers of insulin resistance and diabetes risk.

Although the importance of the obesogenic environment during adult life on metainflammation is now well established, the relative contribution of neonatal nutrition in this biological process is not well understood. In the present study, we used the small litter size model to demonstrate the potent effect of early postnatal overnutrition on adipose tissue inflammation and insulin resistance in adulthood.

## Methods

### Mice

Offspring of C57BL/6 mice (Jackson Laboratories), produced in our mice colony, were used in these studies. Mice were housed under specific pathogen-free conditions and maintained in a temperature-controlled room with a 12 hr light/dark cycle. On post-natal day 1 (P1), litters were adjusted to 7 pups to normalize nutrition. At P3, litters were culled to 3 pups (small litters; SL) containing 2 males and 1 female while normal litters (NL) remained at 7 pups with at least 4 males and 3 females. A subset of male mice were sacrificed at P21, whereas all other males were weaned onto *ad libitum* normal chow and water in pairs matched for the same litter size treatment. At 5 weeks of age, both NL and SL mice were either given 60% high-fat diet (HFD; Research Diets; 90% of fat as lard and 10% as soybean oil) or remained on regular chow (Chow) until sacrifice 12 weeks later (week 17 of life). Animal usage was in strict compliance with and approved by the Institutional Animal Care and Use Committee of the Saban Research Institute of the Children’s Hospital of Los Angeles. Tissues were collected from mice after deep anesthesia with ketamine/xylazine and exsanguination. All efforts were made to minimize suffering throughout the duration of the study.

### Physiological measures

Animals were weighed every two days between P4 and P20 and weekly from P25 through ~P120 (n = 19–25 per group from ≥ 5 litters). At P21, all data are from n = 4–5 per group from ≥ 4 litters, while sample sizes for adult mice are specified below. Epididymal (eWAT) and subcutaneous (sWAT) adipose tissue was collected at P21, and eWAT, retroperitoneal (rWAT), and inguinal (iWAT) fat depots were collected at 17 weeks of age and weighed (n = 9–13 per group from ≥ 6 litters). Insulin tolerance tests (ITT) were performed at 15 weeks of age (n = 4–8 per group from ≥ 4 litters) by an i.p. administration of 0.75U/kg (Humalin R) after a 6-hour fast, and then the blood glucose levels were measured 0, 15, 30, 45, 60, 90, 120 min following insulin challenge. Glucose tolerance tests (GTT) were performed at 16 weeks of age (n = 5–10 per group from ≥ 5 litters) by an i.p. administration of glucose (1.5mg/g bodyweight) after overnight fasting, and then the blood glucose levels were measured 0, 15, 30, 45, 60, 90, 120, and 150 min following glucose challenge. The incremental (and inverted for ITT) area under the glucose curve (iAUC) was calculated using the trapezoidal rule. Total triglyceride (TG) content was assayed in the liver of 17-week-old mice (n = 5–11 per group from ≥ 5 litters) using a Triglyceride Assay Kit (Sigma) as previously described [29].

### Serum measurements

Serum IL-6, MCP-1, and resistin were assayed at 17 weeks of age (n = 4–8 per group from ≥ 4 litters) on a Luminex-MAGPIX using a commercially available multiplex ELISA panel (MADKMAG-71K, EMD Millipore). TNF-α and insulin were assayed using ELISA kits (EMD Millipore).

### Immunohistochemistry and image analysis

Fat pads were sectioned at 1mm thick, fixed in 1% paraformaldehyde and processed for immunofluorescence using standard procedures. Briefly, samples were incubated overnight in primary antibodies, washed, and incubated in secondary antibodies. Samples were counterstained using Hoechst 33342 (Invitrogen) to visualize cell nuclei and immersed in buffered glycerol (pH 8.5). Primary antibodies used were rat anti-F4/80 for CLS (1:1000; Abcam), rat anti-perilipin (1:1000; Sigma) for adipocyte sizing, Armenian hamster anti-CD11c to visualize M1 (1:500; AbD Serotec), and conjugated CD301-Alexa647 (1:200; AbD Serotec) to visualize M2 macrophages. Secondary antibodies were goat anti-rat Alexa 568, anti-rabbit Alexa 488, and anti-hamster Alexa 647 (1:200; Invitrogen). For quantification, images were acquired using a Zeiss LSM 710 confocal microscope system equipped with a 10X or 20X (for P21) objective. Determination of mean size (μm^2^) was measured semi-automatically using the FIJI distribution [30] of Image J software (NIH, ImageJ1.47i). The average adipocyte size and isolated macrophage counts from 3 fields in each mouse was used for statistical comparisons. Adipocyte number per fat pad was calculated based on fat pad weight and adipocyte volume, with the latter derived from the mouse-specific mean and standard deviation of adipocyte diameters obtained from the adipocyte size measurements [31]. The sum total of CLS from 3 fields per mouse were manually counted using Image J analysis software.

### Isolation of stromal vascular cells and flow cytometry

17 week-old mice (n = 7–8 per group from ≥ 4 litters) were perfused with 15 ml of PBS, and one epididymal fat pad was placed in Hank’s Balanced Salt Solution (HBSS; Invitrogen) supplemented with 1% BSA. Adipose tissue was minced and incubated at 37°C in 1mg/ml collagenase (type IV; Worthington Biochemical, Lakewood, NJ). The cell suspension was filtered through a 100 μm filter and centrifuged. The pellet of stromal vascular cells (SVC) was re-suspended in 500 μl of RBC lysis buffer (BioLegend), followed by dilution with PBS containing 1 mM EDTA, 25 mM HEPES and 1% heat-inactivated fetal bovine serum. A minimum of 1x10^5^ cells were aliquoted into single-stain controls, fluorescence minus one controls, and sample tubes, then incubated in Fc Block (rat anti-mouse CD16/CD32) followed by conjugated antibodies. DAPI was used to discriminate live/dead cells. Flow data were acquired on a FACSAria-I (BD Bioscience) and analyzed using Cytobank [32]. FMO controls were used to establish polygonal gates. The following commercially available fluorochrome conjugated antibodies were used: CD45 Apc-Cy7 for leukocytes, CD64 PerCp-Cy5.5 for macrophages, CD11c PE-Cy7 for M1 macrophages (BioLegend, San Diego, CA), and CD301 Alexa 647 for M2 macrophages (AbD Serotec, Raleigh, NC).

### Quantitative real-time PCR

sWAT of P21 and eWAT of 17 weeks-old mice (n = 5–8 per group from ≥ 5 litters) was rapidly dissected and frozen. Total RNA was isolated using the RNeasy Lipid Tissue Kit (Qiagen, Valencia, CA). cDNA was generated using a High Capacity cDNA Reverse Transcription Kit (Applied Biosystems, Carlsbad, CA). Quantitative real time PCR analysis was performed using TaqMan Fast universal PCR Mastermix and recommended primer/probe sets (Life Technologies). mRNA expression was calculated using the 2^-ΔΔCT^ method after normalization with *Gapdh* as an internal control. *Gapdh* levels did not differ between groups. Inventoried TaqMan® Gene expression assays *Il6* (Mm 00446190_m1), *Tlr4* (Mm00445273_m1), *Tnfa* (Mm 00443260_g1), *Itgax* (Mm00498698_m1, *Il10* (Mm00439614_m1), *Arg1* (Mm00475988_m1), *Ccl2* (Mm00441242_m1) and *Gapdh* (Mm99999915_g1) were used. Assays were performed with a Prism 7900HT Sequence Detection System (Applied Biosystems).

### Statistical analysis

Terminal samples at weaning were derived from mice born in different litters, and were therefore independent statistical units analyzed by unpaired T-tests. Terminal samples in adult mice contain data from mice born to the same litter, which results in statistical dependence between siblings; therefore linear mixed models were used to account for within-litter correlation (random intercept for litter) and the within-mouse correlation from repeated-measurements (random intercept for litter + random intercept for mouse + serial correlation) in growth curve, ITT, and GTT analyses. Repeated measurements were analyzed by 3-way ANOVA, while terminal data were analyzed by 2x2 ANOVA. Following a significant interaction (*P*<0.10), post-hoc comparisons between litter sizes were stratified by diet. Variance weights were estimated to mitigate the severe heteroscedasticity caused by the large treatment effect of the HFD. CLS counts were analyzed by generalized estimating equations using a Poisson distribution and the Jackknife estimate of the standard errors [33]. Statistical significance was considered *P*<0.05, and for *post hoc* comparisons, *P* values were adjusted by the Holm-modified Bonferroni correction [34]. Data were analyzed using R 2.15.2 with packages nlme, car, geepack, and phia [35].

## Results

### Small litter rearing causes early rapid weight gain without inducing marked inflammation at weaning

**Fig 1:**
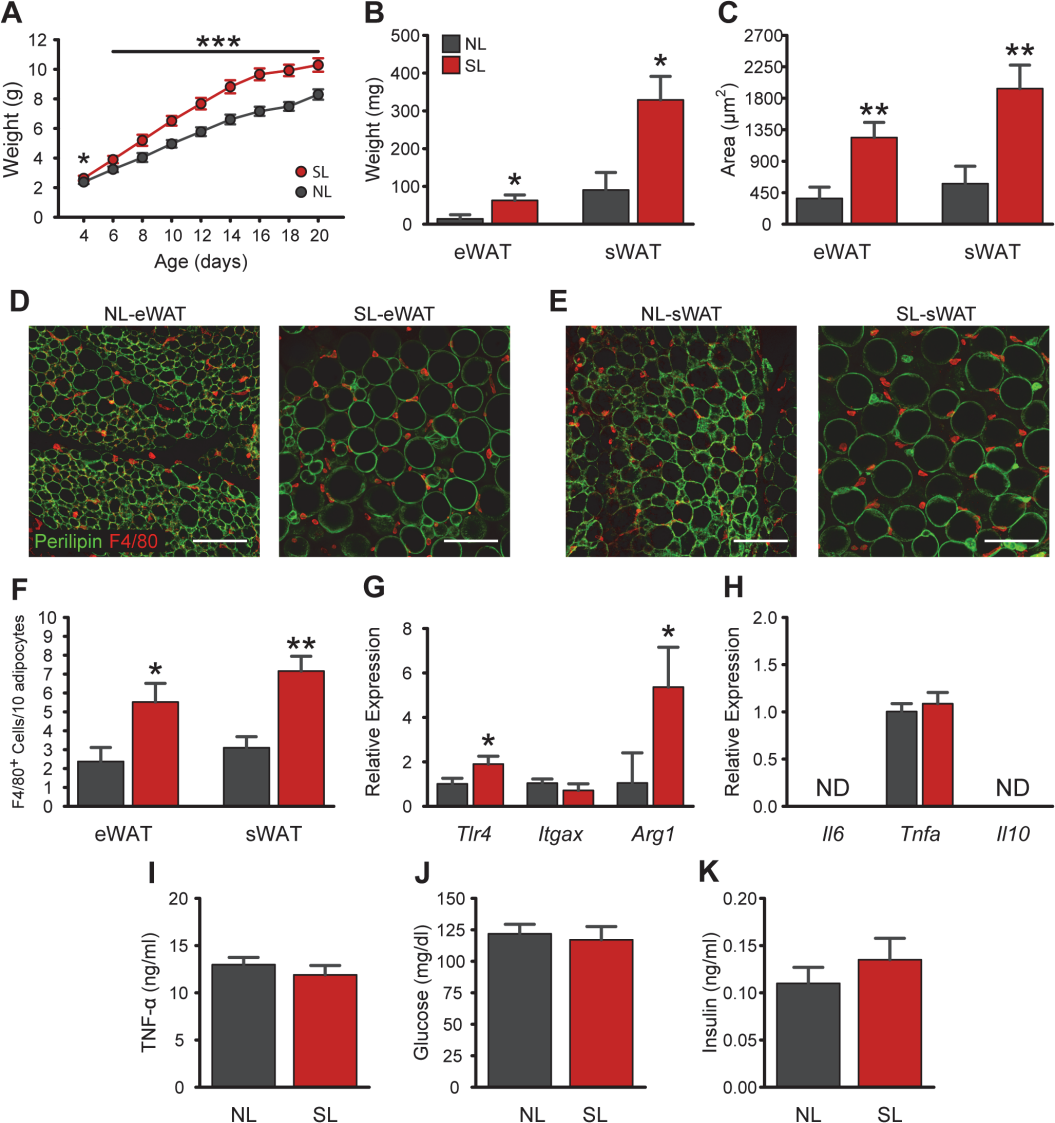
Neonatal overfeeding promotes rapid weight gain and increases adiposity without causing WAT inflammation at weaning. *A*: Pre-weaning growth curves (body weights) of mice raised in normal litters (NL) or small litters (SL) **(** *n* = 19–25 per group from ≥ 9 litters). *B-C*: Mass (*B*) and mean adipocyte areas (*C*) of epididymal (eWAT) and subcutaneous (sWAT) adipose tissue of P21 SL and NL mice **(** *n* = 4–5 per group from ≥ 5 litters). *D-E*: Representative images showing adipocyte morphology (immunostained for perilipin, *green* fluorescence) and F4/80-immunoreactive cells (*red* fluorescence) in eWAT (*D*) and sWAT (*E*) of NL and SL mice at P21. *F*: Quantification of F4/80-immunoreactive cells in eWAT (*D*) and sWAT (*E*) of NL and SL mice at P21 **(** *n* = 4–5 per group from ≥ 4 litters). *G-H*: Relative gene expression of macrophage markers (*G*) and cytokines (*H*) in sWAT at P21. *I-K*: Plasma levels of TNF-α (*I*), glucose (*J*), and insulin (*K*) in NL and SL mice at P21 **(** *n* = 4–5 per group from ≥ 4 litters). *P<0.05 and **P<0.01 versus NL. Scale bar, 100 μm.

To study the consequences of early postnatal overnutrition, we used a mouse model of divergent litter size. As expected, SL rearing was associated with changes in pre-weaning growth, as revealed by a significantly higher body weight in SL compared to NL mice starting at P4 (Fig 1*A*). At weaning, SL pups remained 20% heavier compared to NL pups (P<0.001 at P20; Fig 1*A*). In addition, epididymal (eWAT) and subcutaneous fat (sWat; *i*.*e*., inguinal + paracostal) masses were 4.5-fold and 3.6-fold increased, respectively, in P21 mice compared to control NL animals (Fig 1*B*). This increase in fat mass in weanling SL mice was accompanied by a 3-fold increase in adipocyte area (P<0.01; Fig 1*C*–1*E*). No differences in adipocyte number were detected between NL and SL mice in either eWAT or sWAT (S1 Fig). We next investigated adipose tissue macrophage infiltration using immunostaining for the macrophage marker F4/80. A 2.3-fold increase in the number of F4/80-positive cells was found in eWAT (*P*<0.05) and sWAT (*P*<0.01) of P21 SL mice as compared to control NL mice (Fig 1*D*–1*F*). However, CLS were undetected in both groups. Because obesity induces a phenotypic switch in adipose tissue macrophage polarization from an M2-polarized state to an M1 pro-inflammatory state [23], we also assessed M1- and M2-related gene expression in sWAT of SL and NL at P21. mRNA expression of the anti-inflammatory gene *Arg1* was 5.4-fold increased (Fig 1*G* *; P*<0.05) in SL animals whereas *Il10* gene expression was undetectable (Fig 1*H*). There were no differences in the expression of the proinflammatory genes *Itgax* or *Tnfa*, whereas TLR4 (*P*<0.05) was expressed 2-fold higher in SL mice (Fig 1*G*–1*H*). Consistent with the idea that neonatal overnutrition does not cause marked WAT inflammation at weaning, SL rearing had no effect on random-fed circulating levels of TNF-α, glucose, or insulin (Fig 1*I*–1*K*).

### Neonatal overnutrition exacerbates HFD-induced weight gain and alters fat distribution

**Fig 2:**
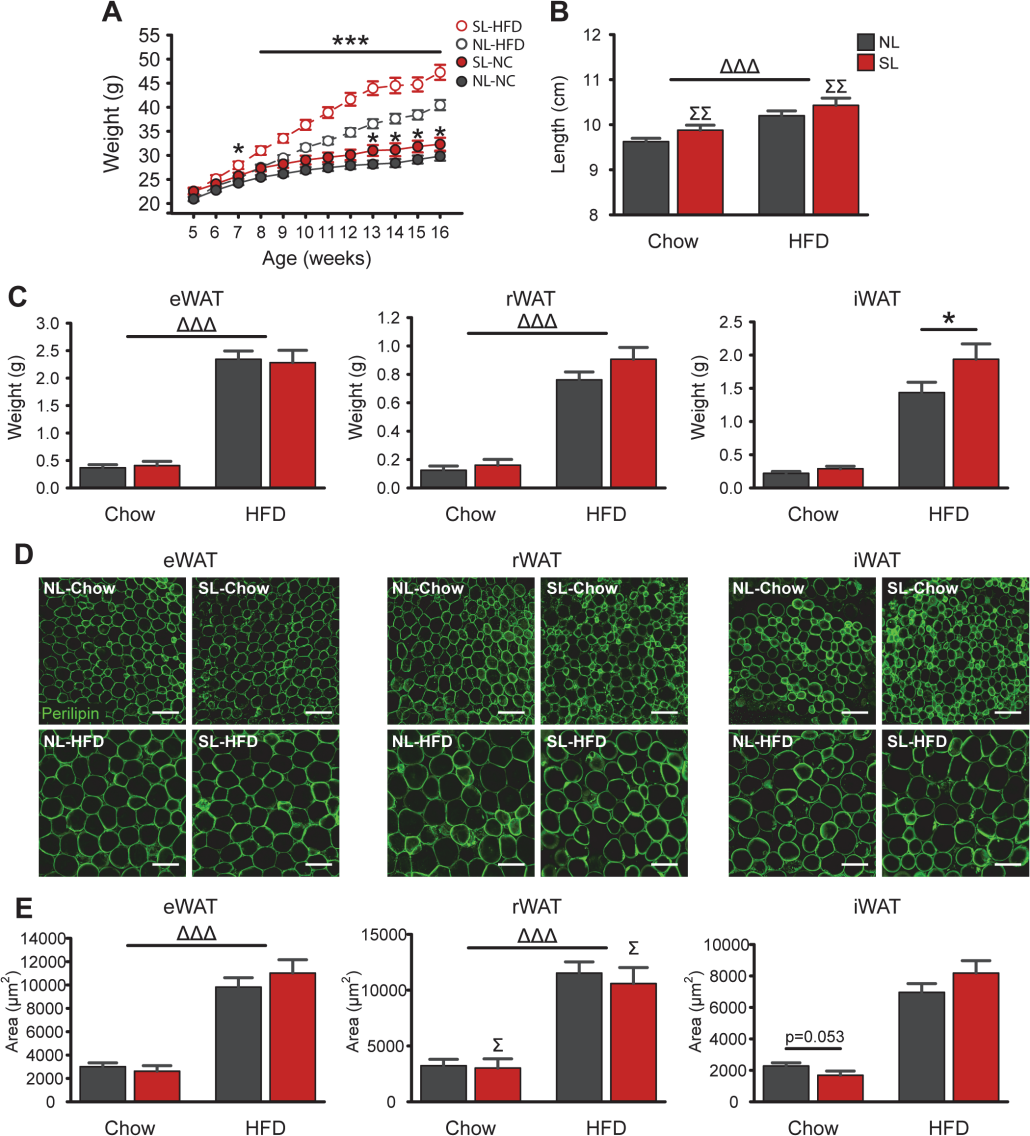
Neonatal overfeeding increases HFD-induced weight gain and alters fat distribution. *A-B*: Post-weaning bodyweights (*A*) and naso-anal length (*B*) of mice raised in normal litters (NL) or small litters (SL) and fed a chow or a high-fat diet (HFD) starting at 5 weeks of age **(** *n* = 9–13 per group from ≥ 5 litters). *C-E*: Mass (*C*) and mean adipocyte areas (*E*) of epididymal (eWAT), retroperitoneal (rWAT), and inguinal (iWAT) adipose tissue in adult SL and NL mice fed a chow or a HFD **(** *n* = 7–8 per group from ≥ 4 litters). Representative images illustrating adipocyte morphology (immunostained for perilipin, *green* fluorescence) in adult NL and SL mice fed a chow or a HFD (*D*). *P<0.05 and ***P<0.001 versus NL matched for diet; Δ *P*<0.05, ΔΔΔ *P*<0.001 for Diet main-effect; Σ *P*<0.05, ΣΣ *P*<0.01 for Litter Size main-effect. Scale bar, 100 μm.

To determine whether neonatal overnutrition programs diet-induced obesity, we fed SL and NL a high-fat diet starting at 5 weeks of age. Body weight was higher in NL-HFD mice compared to chow fed controls as early as 4 weeks after diet manipulation (Fig 2*A*). However, exposure of HFD to SL mice resulted in an even greater increase in bodyweight compared to NL-HFD mice (*P*_Size X Diet X Week_ <0.01; Fig 2*A*). Differences in body weight were detected as early as 2 weeks of high fat feeding (*P*<0.05), and persisted throughout the HFD exposure (*P*<0.05; Fig 2*A*). The elevated bodyweight observed in SL animals was associated with an increase in body length (*P*_Size_<0.01; Fig 2*B*). Moreover, HFD increased eWAT and rWAT weights by nearly 6-fold (*P*<0.001, each) with no additional effect of litter size (Fig 2*C*). However, there was 1.5-fold increase in iWAT weight in SL-HFD animals compared to NL-HFD mice (*P*_Size X Diet_<0.01 and *P*<0.01 for pairwise comparison; Fig 2*C*). High fat feeding also caused adipocyte hypertrophy as revealed by a 3.7-fold, 4-fold, and 3.8-fold increase in adipocyte size in eWAT, rWAT, and iWAT, respectively (*P*_Diet_<0.001, each; Fig 2*D*–2*E*). However, rWAT adipocyte size was reduced by 13% in SL mice compared to NL animals on either diet (*P*_Size_<0.05; Fig 2*D*–2*E*). Adipocyte number was unaltered by diet or litter size in any of the fat pads (S1 Fig).

### Neonatal overnutrition further impairs glucose homeostasis and hepatic steatosis in diet induced obesity

**Fig 3:**
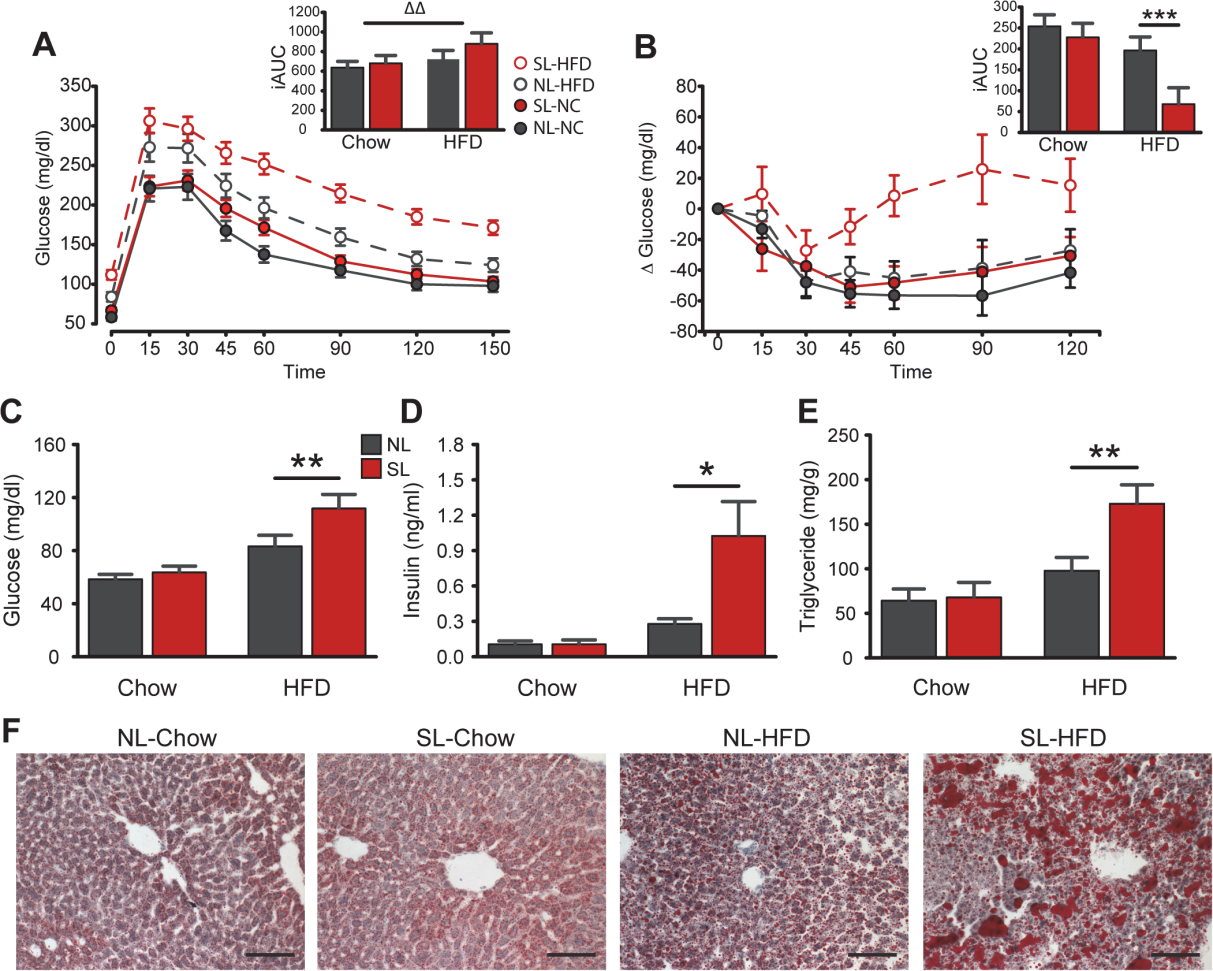
Neonatal overnutrition perturbs glucose homeostasis and causes hepatic steatosis in diet-induced obesity. *A-B*: Glucose and (*A*) insulin (*B*) tolerance tests and incremental area under the curves/inverted incremental area under the curves of adult SL and NL mice fed a chow or a high-fat diet (HFD) (*n* = 4–10 per group from ≥ 4 litters). *C-E*: Plasma levels of glucose (*C*), insulin (*D*), and hepatic triglyceride content (*E*) of adult SL and NL mice fed a chow or a HFD (*n* = 5–11 per group from ≥ 5 litters). *F*: Representative images showing Oil Red-O stain in the liver of adult SL and NL mice fed a chow or a HFD. *P<0.05, **P<0.01 and ***P<0.001 versus NL matched for diet; ΔΔ *P*<0.01 for diet main-effect. Scale bar, 100 μm.

To examine the effect of neonatal overfeeding on glucose homeostasis, we performed glucose (GTT) and insulin (ITT) tolerance tests and measured various fasting parameters in SL and NL mice fed a chow diet or a HFD. Fasting glucose and insulin levels were significantly elevated in SL-HFD mice compared to NL-HFD animals (Fig 3*C*–3*D*). In addition, when exposed to a glucose challenge, SL-HFD mice displayed impaired glucose tolerance as compared to NL-HFD mice across all time points (*P* <0.01; Fig 3*A*). HFD exposure increased the mean iAUC in the GTT (*P*_Diet_<0.003), however, there was only a trend for a higher iAUC in SL mice (*P*_Size_ = 0.067; Fig 3 *A inset*). An ITT was performed to better evaluate whole-body insulin action. During the ITT, blood glucose in SL-HFD mice was statistically significantly higher than NL-HFD animals at 60, 90, and 120 min post insulin injection (*P*<0.001, 0.001, and 0.01, respectively; Fig 3*B*); and the mean ITT-derived iAUC of the SL-HFD group was one-third that of the NL-HFD group (*P*<0.001; Fig 3 *B inset*). In addition, SL animals exposed to HFD displayed a 2-fold increase in the mean hepatic triglyceride content and enhanced hepatic steatosis compared to NL-HFD mice (*P*<0.01; Fig 3*E*–3*F*).

### Neonatal overfeeding triggers HFD-induced adipose tissue inflammation

**Fig 4:**
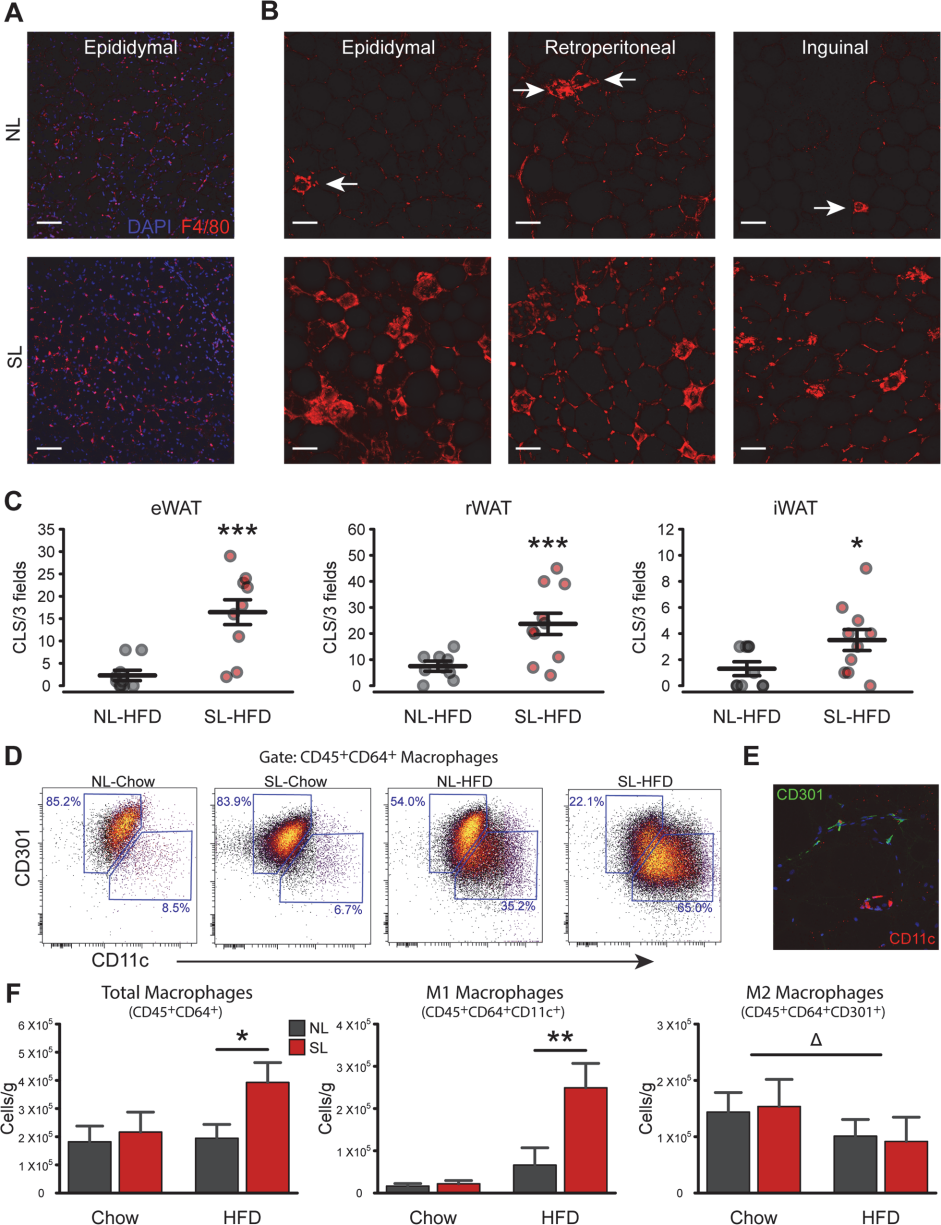
Neonatal overnutrition exacerbates HFD-induced adipose tissue inflammation. *A*: Representative images of F4/80 immunoreactivity (*red* fluorescence) in the epididymal adipose tissue of NL and SL mice fed a chow diet. *B*: Representative images of F4/80 immunoreactivity (*red* fluorescence) in the epididymal, retroperitoneal, and inguinal adipose tissue of NL and SL mice fed a high-fat diet (HFD). *C*: Quantification of crown-like structures (CLS) in the epididymal (eWAT), retroperitoneal (rWAT), and inguinal (iWAT) adipose tissue of adult NL and SL mice fed a HFD (*n* = 9–13 per group from ≥ 6 litters). *D*: Representative scatterplots showing CD301 and CD11c heterogeneity in CD45^+^CD64^+^ macrophages from eWAT of adult NL and SL fed a chow or a HFD. *E*: Histological illustration of antibodies used for flow cytometry. *F*: Quantification of CD45^+^CD64^+^ total macrophages, CD45^+^CD64^+^CD11c^+^ M1 macrophages and CD45^+^CD64^+^CD301^+^ M2 macrophages in eWAT of SL and NL fed a chow or a HFD (*n* = 7–8 per group from ≥ 4 litters). *P<0.05, **P<0.01 and ***P<0.001 versus NL matched for diet; Δ *P*<0.05 for diet main-effect. Scale bar, 100 μm.

**Fig 5:**
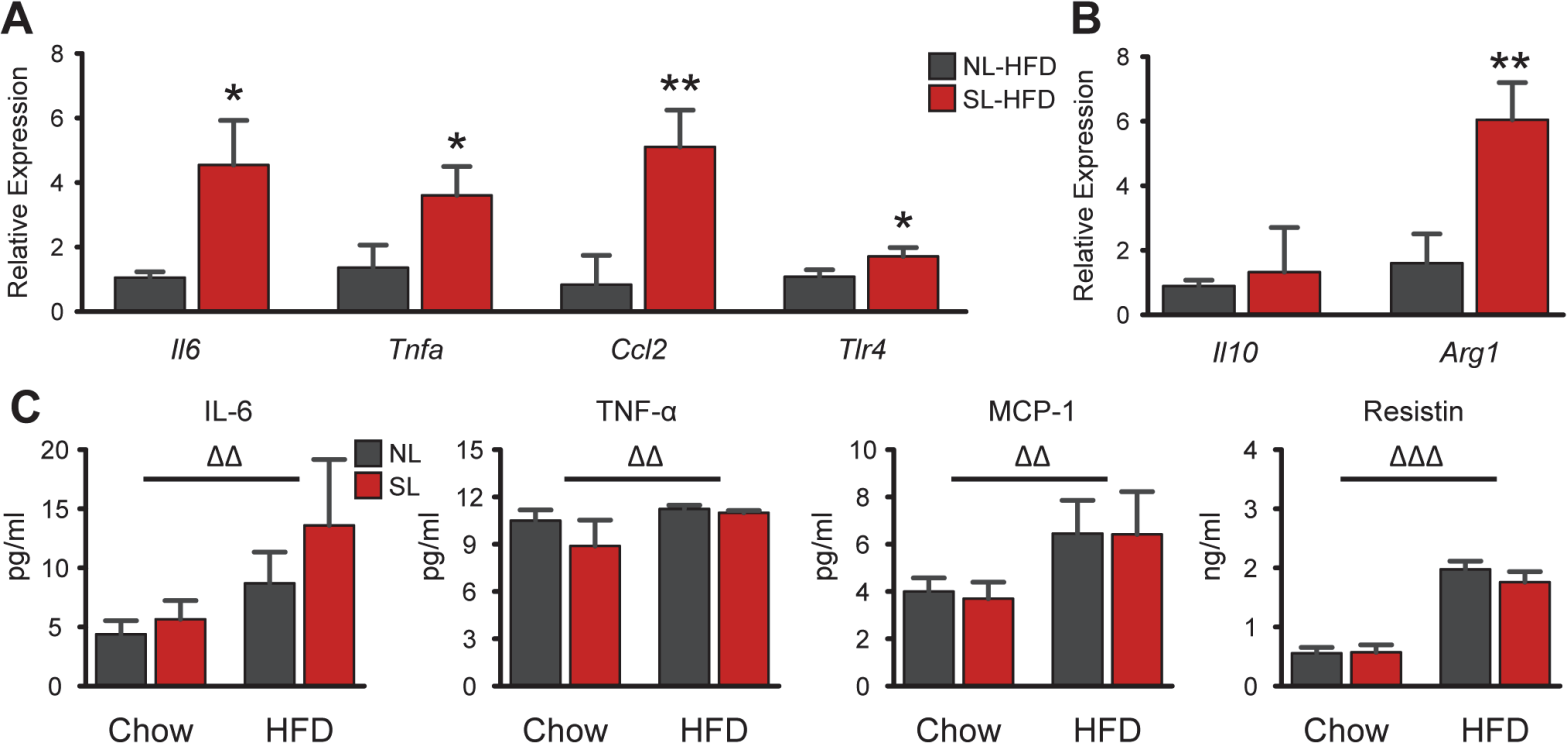
Neonatal overnutrition exacerbates HFD-induced expression of pro-inflammatory genes. *A-B*: Relative gene expression pro-inflammatory (*A*) and anti-inflammatory (*B*) markers in eWAT of NL and SL fed a high-fat diet (HFD) (*n* = 5–8 per group from 5–8 litters). *C*: Serum concentrations of adipokines/cytokines (*n* = 4–8 per group from ≥ 4 litters). *P<0.05 and **P<0.01 versus NL-HFD; ΔΔ *P*<0.01, ΔΔΔ *P*<0.001 for Diet main-effect.

We next evaluated WAT inflammation in various fat pads of adult SL and NL mice fed a chow or a HFD. We first performed F4/80 staining to detect macrophage infiltration and the presence of CLS (Fig 4*A*–4*B*). Consistent with the low-inflammatory potential of low-fat feeding, CLS were rare in the eWAT, rWAT, and iWAT of chow fed NL and SL (Fig 4*A*). Moreover, there was no marked difference in the number of CLS between NL-Chow and SL-Chow (Fig 4A). However, when fed a HFD, SL mice displayed 3–8 times greater CLS counts in eWAT (*P*<0.001), rWAT (*P*<0.001), and iWAT (*P*<0.05) compared to NL-HFD mice (Fig 4*B*–4*C*). To further confirm macrophage infiltration in SL-HFD mice, we quantified total CD45^+^CD64^+^ macrophages in eWAT using flow cytometry. As expected, the total number of macrophages was not different between SL-chow and NL-chow mice (Fig 4*F*). However, on HFD, SL mice displayed twice as many CD45^+^CD64^+^ cells compared to NL-HFD mice (*P*_Size X Diet_<0.01; pairwise *P*<0.05; Fig 4*F*). Histological examination confirms that CLS predominately contain CD11c^+^ M1 macrophages, while CD301^+^ macrophages are identified as more diffuse isolated cells (Fig 4*E*). To better characterize the phenotype of adipose tissue macrophages, we next sorted CD45^+^CD64^+^ macrophages based on heterogeneous expression of CD11c and CD301. The number of CD11c^+^ macrophages was 3.8-fold higher in SL-HFD mice compared to NL-HFD (*P*<0.01; Fig 4*F*). In contrast, the number of CD301^+^ macrophages was reduced by 40% in the HFD groups (*P*_Diet_<0.01), with no additional effect of litter size (Fig 4*F*). These findings confirmed that the increased number of macrophages observed in the eWAT of SL-HFD mice is attributed to an increased number of M1 macrophages. Consistent with these findings, mRNA expression of pro-inflammatory genes, such as *Il6*, *Tnfa*, and *Ccl2* (MCP-1) were up-regulated by 4.5 (*P*<0.05), 3.6 (*P*<0.05), and 5.1 (*P*<0.01) times, respectively, in eWAT of SL-HFD compared to NL-HFD mice (Fig 5*A*). Similar to the P22 animals, *Tlr4* expression in SL-HFD was 1.7 times that of NL-HFD (*P*<0.05; Fig 5*A*). In addition, *Arg1* mRNA expression was 6-fold increased in SL-HFD animals (*P*<0.01), but surprisingly, *Il10* mRNA expression was not different between SL-HFD and NL-HFD mice (Fig 5*B*). Similarly, circulating concentrations of IL-6, TNF-α, MCP-1, and resistin were 2-, 1.1-, 1.7-, and 3.3-fold increased in HFD animals compared to chow fed mice (*P*_Diet_<0.001; Fig 5*C*). However, there were no additional effects of litter size on these circulating cytokines or resistin (Fig 5*C*).

## Discussion

Epidemiological and animal studies have indicated that changes in growth and nutrition during early life can have lasting effects on adult metabolism. In the present study, we employed a well-established animal model of divergent litter size to study the role of early postnatal overnutrition on adipocyte inflammation and metabolic programming. Despite the early rapid weight gain, SL rearing alone did not cause WAT inflammation at weaning or in adults when fed regular chow. However, SL mice were sensitized to greater obesity-induced macrophage recruitment and cytokine signaling in WAT, as well as greater ectopic fat deposition and impaired glucose homeostasis. Importantly, all of these differences occurred despite comparable levels of visceral adiposity between NL-HFD and SL-HFD groups. These experiments demonstrate that early postnatal nutrition influences the inflammatory phenotype of adipose tissue in diet-induced obesity.

Litter size has been known to influence weight gain in rodents for nearly a century [36]. The accelerated growth of SL pups from selectively culled litters is primarily attributed to increased energy intake of the suckling pups [37], and therefore provides an accurate model for perinatal programming by early overnutrition. Confirming the importance of perinatal nutrition, numerous studies have shown that SL rodents maintain increased fat mass and develop components of the metabolic syndrome in adulthood, despite being fed regular chow [13,18,38]. Several studies have further shown that litter size reduction causes greater sensitivity to the metabolic consequences of diet-induced obesity, including greater insulin resistance and hepatic steatosis [16,19,20]. Boullu-Ciocca et al. correlated the exaggerated metabolic impairments to enhanced WAT glucocorticoid signaling [20], while a more recent report by Liu et al. showed further impairments in muscle and WAT insulin signaling [19]. Both studies demonstrate higher cytokine gene expression in WAT from SL-HFD compared to NL-HFD, but these findings were confounded by increased fat mass and adipocyte size in the fat pads of interest. Our study confirms and extends these reports in several important ways. First, we employed several different techniques, including flow cytometry, to comprehensively characterize WAT inflammation and macrophage recruitment. Second, using 12 weeks of a 60% HFD in the C57BL/6 mouse strain, a common model for metainflammation studies, we demonstrated that SL rearing exacerbates inflammation largely independent of differences in adiposity. Finally, by examining NL and SL mice at weaning, we showed that the increased propensity to HFD-induced inflammation in adulthood is not attributed to an early development of inflammation. Together, these findings implicate adipose tissue inflammation as an important contributor to the metabolic programming induced by perinatal overfeeding.

WAT inflammation is characterized by changes in numerous leukocyte populations, particularly macrophages, and increased production of inflammatory cytokines [39]. Previous studies have shown that ablation of CD11c^+^ cells [25] or reduction of their cytokine production [28] mitigates insulin resistance in obesity, suggesting that at least part of the metabolic impairment we observed in SL-HFD mice is attributed to the large increase in M1 macrophage recruitment and subsequent cytokine production. Proinflammatory cytokines from the adipose tissue of SL-HFD could enter the circulation and directly cause insulin resistance in muscle or liver [40,41]. However, circulating concentrations of IL-6, TNF-α, and MCP-1 were comparable between NL-HFD and SL-HFD, suggesting that this was not the primary mechanism. On the other hand, macrophage-derived cytokines can induce adipocyte insulin resistance through paracrine signaling [26], which could impair adipocyte function, resulting in ectopic fat deposition and lipid-induced insulin resistance [42]. Our findings showing elevated WAT cytokine gene expression and hepatic triglyceride accumulation are consistent with this hypothesis.

As early as 3 weeks of life, SL mice already displayed greater fat mass and adipocyte hypertrophy compared to NL mice. Yet the juvenile SL mice did not develop overt WAT inflammation, as demonstrated by the absence of CLS and normal *Itgax* (CD11c) expression, but instead, demonstrated an M2 phenotype, with higher numbers of isolated macrophages and an increase in *Arg1* expression. Furthermore, minimal differences in adiposity between NL-Chow and SL-Chow mice were observed in adulthood, suggesting that accelerated fat deposition, rather than overall increased adiposity, with perinatal overnutrition may alter the sensitivity of adipose tissue to obesity-induced inflammation. The absence of an overt inflammatory phenotype in weanling SL mice excludes premature development of inflammation as the explanation for greater leukocyte infiltration in SL-HFD mice in adulthood. Several other mechanisms, however, could explain the programmed response to adipose tissue inflammation.

The accelerated fat gain during development could induce pathological remodeling of the extracellular matrix that could persist into adulthood. Fibrosis and dysfunction of adipose tissue, including visceral and subcutaneous depots, is an important contributor to inflammation and insulin resistance [43]. The observed increase in M2 macrophages in SL adipose tissue at weaning, and increased CLS formation in the generally protected subcutaneous fat of adult SL-HFD mice, are consistent with this hypothesis. Hyperinsulinemia was recently shown to mediate the programming effect of early postnatal overnutrition on impaired glucose homeostasis [15]. An interesting question is whether this excessive insulin signaling is also responsible for the increased inflammatory sensitivity in adipose tissue of SL offspring. Furthermore, persistent alterations in the responsiveness of adipose tissue to fatty acids [44] or differential development of the gut microbiome [45] could potentially link SL rearing to later adipose tissue inflammation, and are consistent with the increased *Tlr4* expression we observed in WAT from SL mice at weaning. Future experiments will be required to test these hypotheses and identify other potential mechanisms.

Epidemiological data confirms the relevance of early life obesity to later metabolic health in humans. Obesity rates have increased in children younger than 5 years of age [46], and this excessive early growth is associated with greater propensity for obesity [47] and insulin resistance [48] in later life. Similar to the discordance in inflammation seen between NL-HFD and SL-HFD mice in the current study, our group [49], as well as others [9], has shown that macrophage infiltration in WAT is associated with greater insulin resistance among similarly obese patients. However, the factors that determine whether an obese patient subsequently develops this inflammation are poorly understood. Given the profound inflammatory and metabolic differences between NL and SL mice on HFD, our findings offer proof of principle that perinatal nutrition may be an important environmental contributor to metabolically abnormal obesity, possibly by exacerbating WAT inflammation.

In conclusion, our study shows that SL rearing results in greater fat mass accretion before weaning without inducing early-onset WAT inflammation. When exposed to an obesogenic environment, mice raised in a SL displayed much greater macrophage recruitment and cytokine gene expression compared to NL mice, independent of differences in visceral fat. This greater propensity to develop adipose tissue inflammation paralleled more severe hepatic steatosis and impairments in glucose homeostasis. Together, these data suggest that the early postnatal environment may be a critical modifier of adipose tissue inflammation and insulin resistance in diet-induced obesity.

## Supporting Information

S1 Fig Neonatal overfeeding did not alter adipocyte number in fat pads from weanling nor mature animals fed chow or high fat diet. *A*: Number of adipocytes in epididymal (eWAT) and subcutaneous (sWAT) fat pads from P21 mice raised in normal litters (NL) or small litters (SL) (*n* = 4–5 per group from ≥ 4 litters). *B*: Number of adipocytes for the epididymal (eWAT), retroperitoneal (rWAT), and inguinal (iWAT) depots from mature NL and SL mice remaining on normal chow (Chow) or high fat diet (HFD) for 12 weeks (*n* = 7–8 per group from ≥ 4 litters).
(TIFF)
