# Supplementary figures and images for: Perinatal Overnutrition Exacerbates Adipose Tissue Inflammation Caused by High-Fat Feeding in C57BL/6J Mice
Source: PLoS One. 2015 Apr 2;10(4):e0121954. doi: 10.1371/journal.pone.0121954 (PMC4383546; doi:10.1371/journal.pone.0121954)

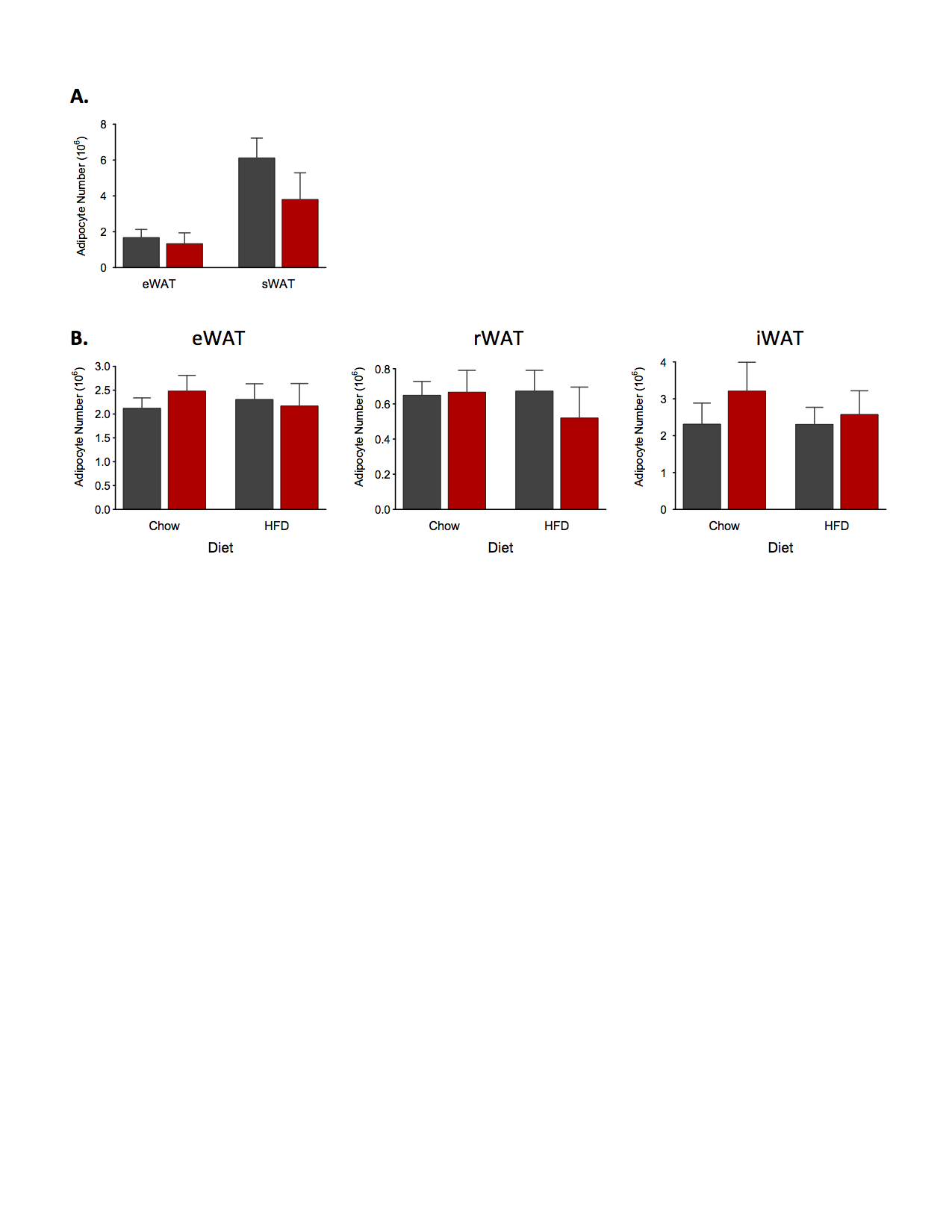

Supplement: S1 Fig — A: Number of adipocytes in epididymal (eWAT) and subcutaneous (sWAT) fat pads from P21 mice raised in normal litters (NL) or small litters (SL) (n = 4–5 per group from ≥ 4 litters). B: Number of adipocytes for the epididymal (eWAT), retroperitoneal (rWAT), and inguinal (iWAT) depots from mature NL and SL mice remaining on normal chow (Chow) or high fat diet (HFD) for 12 weeks (n = 7–8 per group from ≥ 4 litters). (TIFF) [file pone.0121954.s001.tiff]
